# Does diversifying crop rotations suppress weeds? A meta-analysis

**DOI:** 10.1371/journal.pone.0219847

**Published:** 2019-07-18

**Authors:** David Weisberger, Virginia Nichols, Matt Liebman

**Affiliations:** Department of Agronomy, Iowa State University, Ames, Iowa, United States of America; USDA Agricultural Research Service, UNITED STATES

## Abstract

Over the past half-century, crop rotations have become increasingly simplified, with whole regions producing only one or two crops in succession. Simplification is problematic from a weed management perspective, because it results in weeds’ repeated exposure to the same set of ecological and agronomic conditions. This can exacerbate weed infestations and promote the evolution of herbicide resistance. Diversifying crop rotations through addition of crop species and their associated managements may suppress weeds and reduce selection pressure for herbicide resistance by altering stress and mortality factors affecting weed dynamics. Here we report the results of a meta-analysis using 298 paired observations from 54 studies across six continents to compare weed responses due to simple and more diverse crop rotations. We found diversifying from simple rotations reduced weed density (49%), but did not have a significant effect on weed biomass. We investigated the effect of management practices, environmental factors, and rotation design on this effect. Diversification that increased the variance around crop planting dates was more effective in suppressing weeds than increasing crop species richness alone. Increasing rotational diversity reduced weed density more under zero-tillage conditions (65%) than tilled conditions (41%), and did so regardless of environmental context and auxiliary herbicide use. Our findings highlight the value of diversifying crop rotations to control weed populations, and support its efficacy under varied environmental conditions and management scenarios.

## Introduction

Weed management is an essential part of crop production. Crop yield reductions from weeds average 40% on a global scale, and costs associated with management inputs represent a major economic expense for farmers [1, 2]. Broadly speaking, many major agricultural regions produce only one or two economically-important crops [3]. This reduction of crop rotational diversity has been largely due to the adoption of herbicides and herbicide-resistant crops [4, 5]. While the shifts in crop and weed management practices have been generally effective in controlling weeds and minimizing labor costs, heavy reliance on herbicides has led to adverse environmental and human-health outcomes and an expedited development of herbicide-resistant weeds [6–13]. Conversely, increasing cropping diversity can subject weeds to a greater number of stress and mortality factors [14, 15], and may be critically important for addressing threats from herbicide resistance [12, 14, 15].

Although qualitative and semi-quantitative reviews have described the weed-related effects of crop rotational diversity [15, 16], a quantitative analysis of its efficacy has been lacking. Here, we present the results of a meta-analysis conducted with an extensive data set derived from field studies, representing the first quantitative synthesis of peer-reviewed literature concerning the effects of diversifying crop rotations on weeds in agroecosystems. Our goal was to answer the following questions: (a) Compared to simple rotations of one or two crops, do diverse rotations suppress weeds? (b) What is the magnitude of the suppression if it occurs? (c) Under what conditions is the suppression most pronounced? We quantified weed responses using weed density, an important metric for understanding demographic trajectories, and weed biomass, a proxy for the competitive effect of weeds on crops.

## Materials and methods

### Literature search

**Fig 1:**
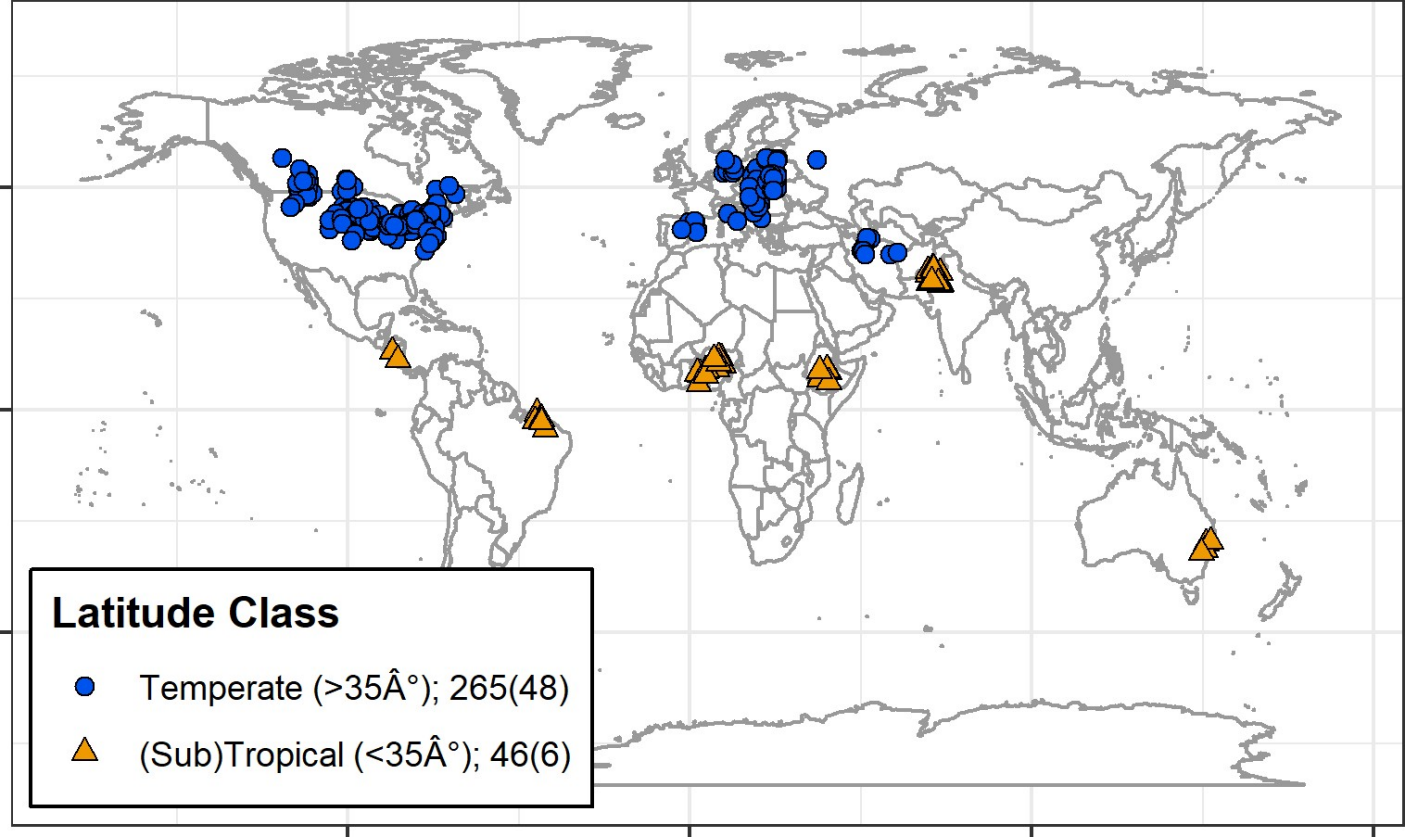
Map of meta-analysis study locations. Temperate (blue circles, >35° latitude) and sub-tropical/tropical (orange triangles, <35°) study locations with number of data points and number of studies (in parentheses) for each latitude class.

We conducted a systematic search of relevant literature using ISI Web of Knowledge (WoS, available online). A search was conducted in January 2018, using the following Boolean string: ("crop rotation*" OR "crop sequence*" OR "multiple cropping*" OR “organic”) AND (management* OR control*) AND ("weed biomass*" OR "weed density*" OR "weed seed bank*") NOT (orchard* OR vineyard* OR agroforestry*). No geographical or language restrictions were applied to the screening process, and the search period in ISI WoS, in both cases, was “All years” (i.e. 1864–2018). This search resulted in a total of 1276 articles. An additional 10 records came from the authors' collections of relevant literature. After removing duplicates, this resulted in a total of 885 articles, whose titles and abstracts were screened for eligibility. Articles were screened using guidelines [17] based on the following criteria: (a) articles were published in peer-reviewed journals (as opposed to book chapters or conference abstracts), (b) articles were based on studies conducted with structured experimental designs in a field setting, (c) articles reported one or both of the following response variables: weed biomass and weed density, (d) studies included a simplified rotation (control) and a diversified rotation (treatment). This produced 102 full-text articles which were further screened. We then eliminated studies where rotational diversity was enhanced only by inclusion of a non-harvested cover crop. While cover crops increase the diversity of crop rotations, our goal in this study was to examine rotations that increased diversity via harvested crops; other meta-analyses have looked specifically at cover crop-weed interactions [18, 19]. No screening was done with respect to the type of crop harvested (grain, fruit, tuber, root, and forage biomass were included). Less than 10% of the studies that we reviewed reported crop yields, and due to this small and un-representative sample size we chose not to include crop yield in our analyses. This resulted in 54 studies being selected for our database. A full list of included publications and the PRISMA flow diagram [20] detailing the literature search process is provided (S1 Text, S1 Fig, S1 Table). A map showing the geographic locations of studies included in our dataset is presented in Fig 1.

### Data extraction and processing

Data was extracted from text, figures (GetData graph digitizer, http://getdata-graph-digitizer.com/), and datasets acquired through personal communication when necessary. All data manipulation, analysis and graphics were completed with the R (version 3.5.1) [21] packages readxl [22] and tidyverse [23]. A complete description of the extraction process and resulting information is provided (S2 Text). Weed response variables were biomass (g m^-2^) or density (plants m^-2^). The resulting raw dataset (n = 891) is available through Iowa State University’s DataShare [24].

Comparisons of simple versus diverse rotations were determined on a per-paper basis using the number of species present in the rotation as a guide. Within studies, this resulted in the following rotations identified as simple: (a) monocrop (1 species, n = 265), (b) monocrop with alternating years of fallow (1 species, n = 19), and (c) a two-year rotation (2 species grown over two years, n = 63). Continuously growing one crop or two crops in succession (a and c) are salient examples of simple rotations within contemporary agricultural production. Intermittent fallow systems (b) are a form of a simple rotation that is commonplace in arid regions worldwide [5, 25]. If more than one diverse rotation was present in the study, the simple rotation was compared to each diverse rotation separately. Our classification scheme resulted in a total of 64 comparisons for weed biomass and 247 for weed density.

For each comparison, we used the extracted information to create nine factors that could potentially influence the effect of crop diversification on weed dynamics. These included seven categorical variables: (a) latitude class (temperate vs. sub-tropical and tropical), (b) tillage regime (tilled vs. zero-tillage), (c) use of herbicides (yes vs. no), (d) use of fallowing in the simple rotation (yes vs. no), (e) use of a perennial in the diverse rotation (yes vs. no), (f) diversifying from a monoculture (yes vs. no), and (g) the unit of weed measurement (single species vs. sum of multiple species). We also assessed the influence of two continuous variables: the ratio of the number of species in the diverse rotation to the simple rotation and the difference in the coefficient-of-variation of months between planting operations in the diverse versus the simple rotation [26].

### Data analysis

Eleven studies reported both weed density and biomass. Using the raw dataset [24], the Spearman rank correlation (*ρ*) between weed density and weed biomass (n = 236) was calculated in base R [21] using the cor and cor.test functions. In nine comparisons either the treatment or control value was zero. These points were removed from the analysis, similar to Verrett et al. (2017) [27], as adding an arbitrary value can result in comparisons changing from negative to positive or produce unrealistic effect sizes. The response of weed biomass or density to crop rotational diversity was represented by the ratio of the weed response in the diverse rotation to the response in the simple rotation [28–30]. Only nine of the 54 studies reported variances in some form, so we used non-parametric weighting based on sample sizes [29].

To identify the factors with the strongest effects on weed density, we fit a generalized boosted regression tree (BRT) model [31] with the nine modifiers as predictors using the R package *gbm* [32] and *caret* for model tuning [33]. For this model, we eliminated comparisons with incomplete moderator information (n = 41 removed). We calibrated the model to minimize the bootstrapped root-mean-square-error (RMSE). The final model was fit to the data (n = 206) using model parameters of 0.01 shrinkage, 3 node tree depth, and 200 trees with a Gaussian distribution. Variable importance was quantified using the relative influence metric [34]. This model was not fit to weed biomass responses because of insufficient data points with fully defined variables (n = 55).

To estimate effect sizes, we took the natural log of the response ratio and fit a linear mixed-model in R using the lme4 package [35], with study as a random effect and weighting as described above. A separate model was fit to each response variable. Initial analyses were conducted without moderators to assess overall effects. A potential weakness of meta-analyses is that it relies on published literature, which may bias results towards significance. To estimate the robustness of our results against publication bias, we calculated the Rosenthal fail-safe number [36] using the metafor package [37].

To evaluate the effect of moderators identified as important by the BRT, mixed-effect models were fit for responses using each moderator as a single fixed effect, study as a random effect, and non-parametric weighting. Significance of results was calculated using the emmeans package [38], estimating and comparing mean values to zero using the lsmeans function, and comparing them to each other using the contrast function with Satterthwaite degrees of freedom. Results were converted to a percentage change in weed response in diverse rotations relative to simple rotations.

To ensure the robustness of our results, we tested the sensitivity of mean effect sizes to included studies by performing a leave-one-out analysis [30, 39], using the same procedure described above to estimate means and confidence intervals for each subset of data. Based on the sensitivity analysis, we found results were consistent at a significance level of p < 0.01, and thus chose this threshold to assign significance. Estimates and 99% confidence intervals were back-transformed and expressed as a percentage change from the control (simple rotation). Statistical summaries are provided in S1 Tables. All R code and data related to the analyses described above can be found in a Github repository (https://github.com/vanichols/Weisberger-et-al-2019).

## Results and discussion

**Fig 2:**
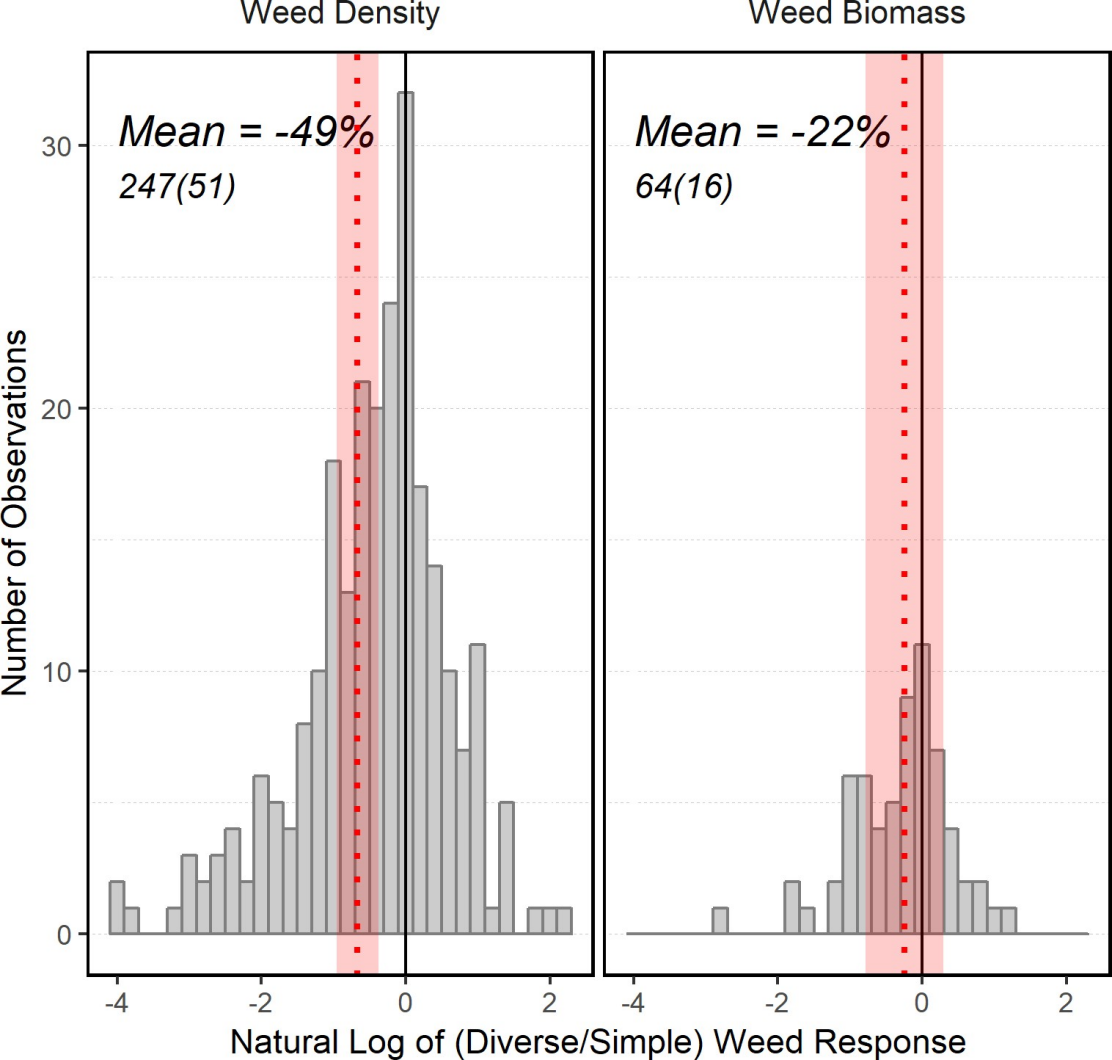
Histogram of weed response (weed density and biomass) to diversifying from a simple rotation. Means (dotted lines) and standard errors of the means (shaded areas) on a natural log scale, mean value on a percent change scale, total number of observations, and number of studies (parentheses).

Diversification of crop rotations significantly reduced weed density compared to simple rotations (Fig 2), with a mean reduction of 49% (n = 247, p < 0.001). In contrast, the effect of diversification on weed biomass was weaker (-21%) and non-significant (n = 64, p = 0.22). The fail-safe analysis indicated the significant effect observed for weed density was robust against publication bias; at least 12,905 non-significant comparisons would have to have remained unpublished during the time frame to negate our findings.

**Fig 3:**
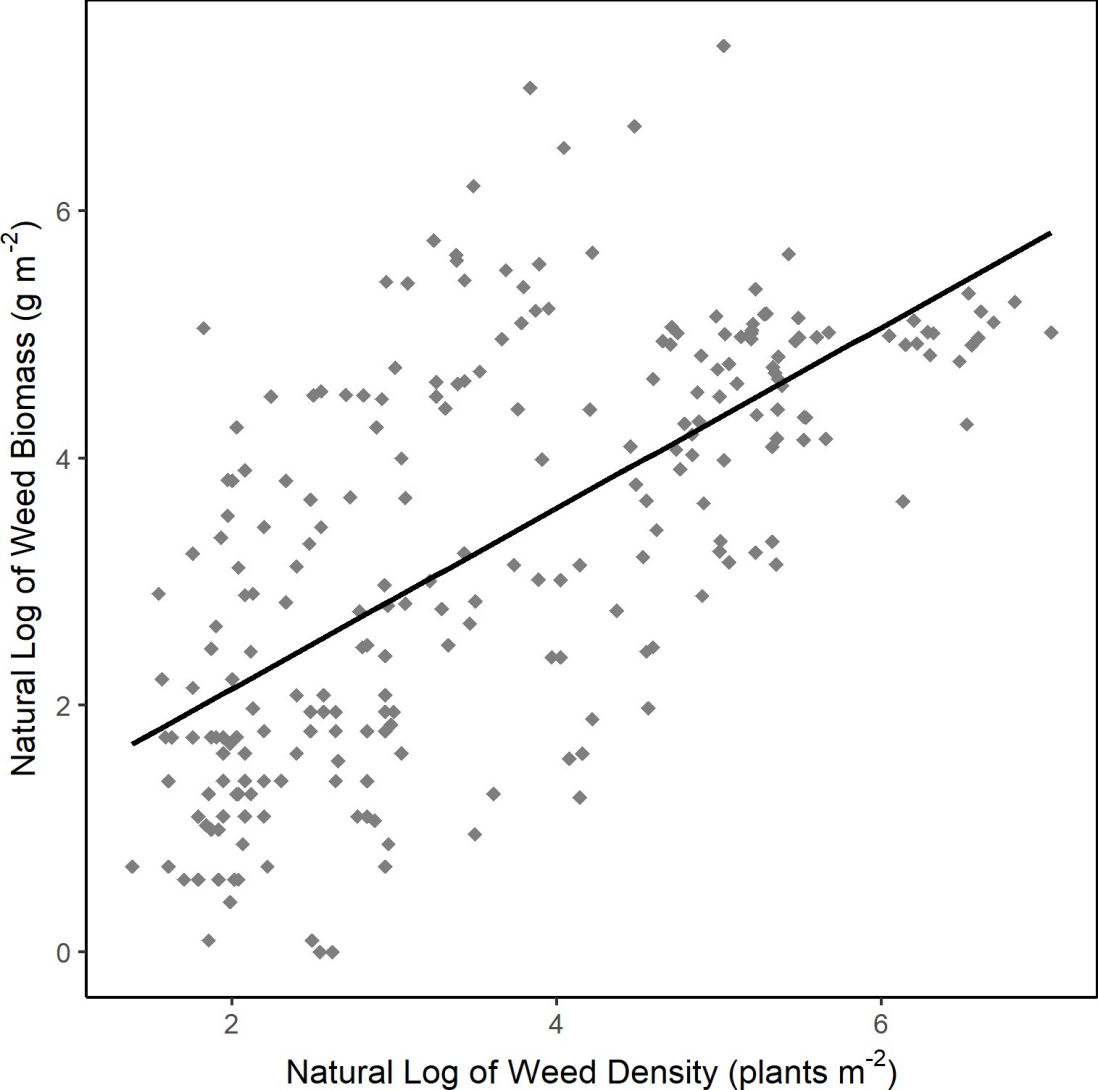
Relationship between weed biomass and weed density (natural log scale) for studies (n = 11) that reported both values. Line represents Spearman correlation fit.

When looking at effect estimates based on studies available in a given year, the estimate for weed density plateaued over time and had sufficient points for high precision (S2 Fig). The estimated response of weed biomass did not have the same precision as density due to the lower number of studies, but leveled off. This suggests that diverse rotations may indeed reduce weed biomass compared to simple rotations, but that the smaller number of studies reporting weed biomass limited our ability to detect a significant effect. In studies that measured both response variables, weed biomass was correlated with weed density (Fig 3, *ρ* = 0.67, p < 0.001). This indicates that reductions in weed density were not associated with compensatory growth, and that lower weed densities equated to lower weed biomass.

**Fig 4:**
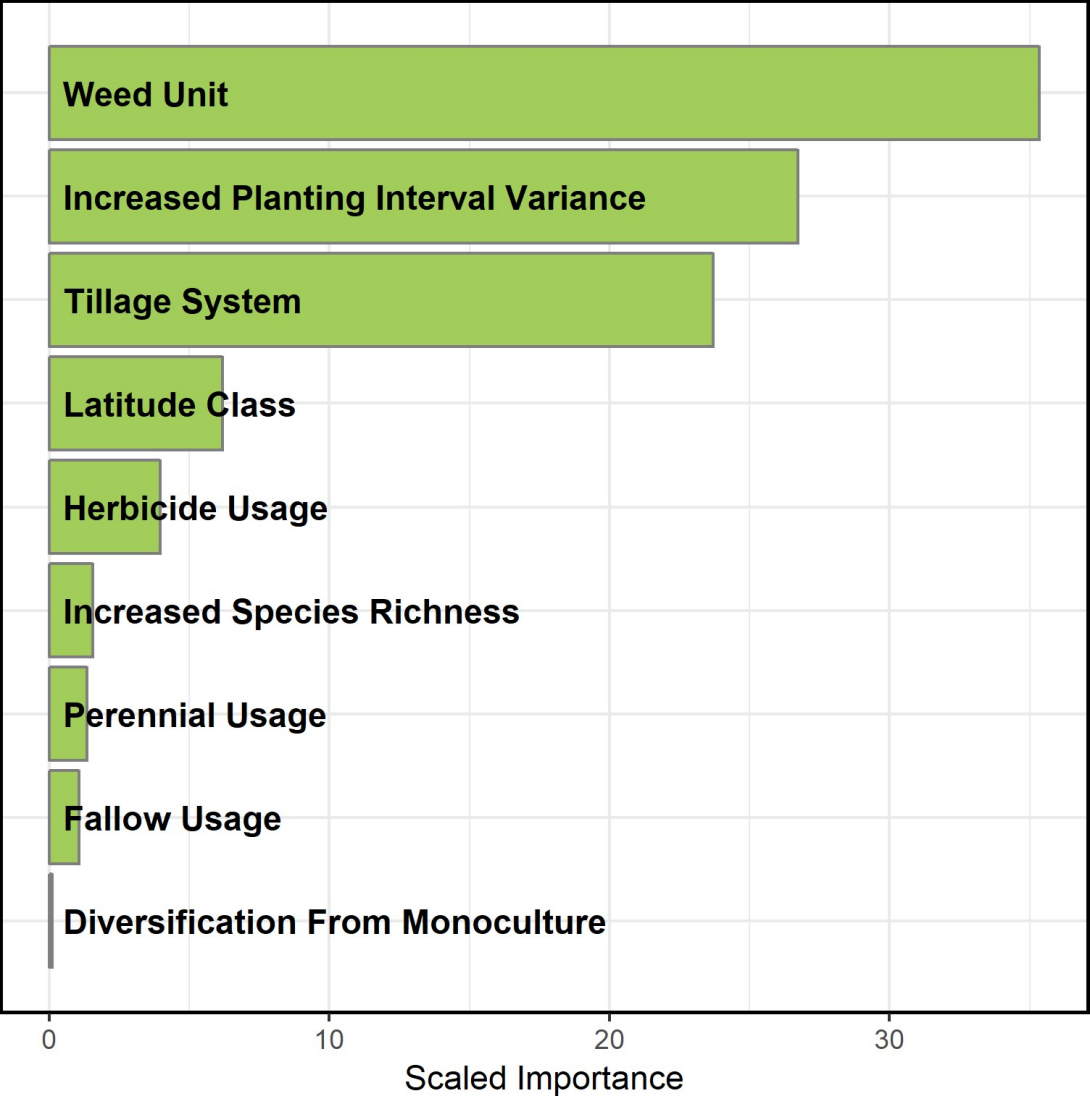
Scaled importance of nine predictors for weed density response to increased rotational diversity. Variable importance derived from a generalized boosted regression tree.

The BRT analysis demonstrated that three moderators were most important in describing the response of weed density to rotational diversification: (a) weed measurement unit, (b) increased planting-interval variation, and (c) tillage system (Fig 4). The remaining six moderators had small importance, with a combined contribution of <15%.

The importance of temporal variance around crop planting dates (Fig 4) agrees with the assessment of Gaba et al. (2014) [26], who suggested that the effects of different cropping systems on weed dynamics could be captured by quantifying the magnitude of changes in sowing dates between successive crops. Consequently, much of the rotation diversification effect on weed density that we observed (Fig 2) is likely linked to associated increases in heterogeneity in the timing of disturbance events, such as planting and herbicide application, as well as temporal variation in periods of crop growth and resource capture. Our results show these complex changes can be captured in the simple metric of variation in intervals between planting activities. In contrast, the relative number of crop species in diversified versus simple rotations was relatively unimportant for explaining weed suppression (Fig 4). Thus, the functional characteristics of a given rotation system, including temporal patterns of disturbance and resource capture, were more important in determining weed density than was crop species richness *per se*.

**Fig 5:**
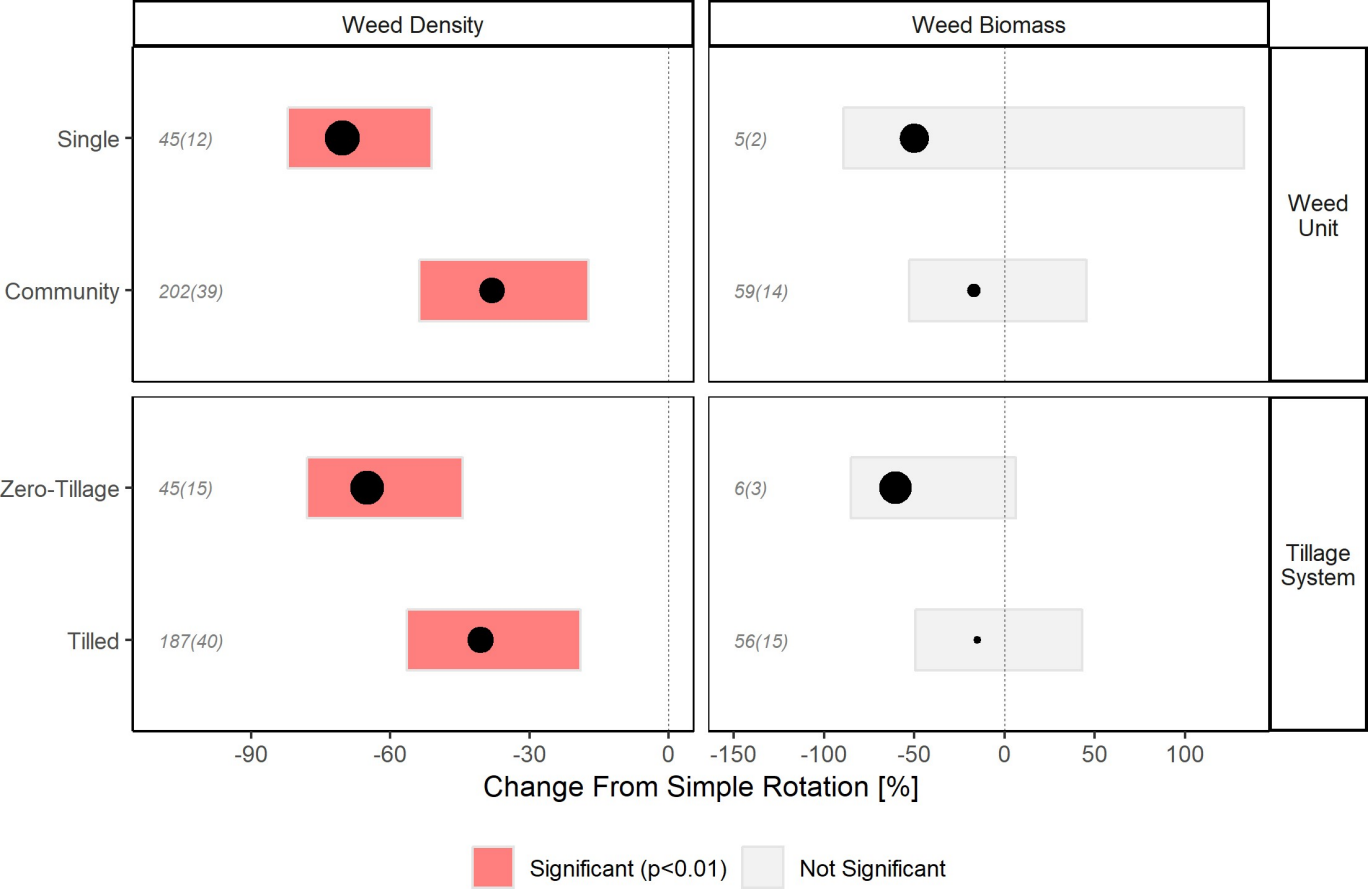
Weed density and biomass responses to increased rotational diversity as affected by weed measurement unit and tillage system. Mean values (point sizes scaled to absolute values), 99% confidence intervals, and number of observations (italics) and number of studies (italics, parentheses), dotted line represents no change, note different x-axis scales.

The importance of the two categorical variables, weed measurement unit and tillage system, is demonstrated through differences in weed suppression between groups within them (Fig 5).

Suppression of weed density was greater (p = 0.001) in diverse rotations where individual weed species were measured (-70%, n = 45) compared to those where multiple weed species were measured together (-38%, n = 202). This could represent a biological phenomenon, or simply be an artifact of study designs. Studies that evaluate management options for problematic single species often focus on practices that are intended to provide effective control of those species specifically, which would result in the relatively larger reductions. Alternatively, due to the increased range of stress and mortality factors introduced by diverse rotations, community compositions may shift to include more species at lower total densities. Recent work has suggested that shifts in weed community composition may be an important indicator of weed management systems that do not place constant uniform selection pressures on weeds [40], which is supported by our results.

Tillage system was highly important in predicting the weed density response to rotational diversification (Fig 4). We found that the effect of crop diversification on weed density was stronger (p = 0.003) for zero-tillage systems (-65%, n = 45) compared to tilled systems (-41%, n = 187) (Fig 5). Tillage can have a major impact on the size and structure of weed communities. It does so by altering the vertical distribution of seeds in the soil, and physically terminating both germinated seeds and emerged plants [41–43]. Across all rotations in our data set, mean weed density in zero-tillage systems was lower than in tilled systems, however the effect of rotational diversification was amplified by zero-tillage management. This agrees with literature reviews that suggest that weed management in reduced or zero-tillage systems may benefit from rotation diversification [44, 45]. Other reviews have shown that diversifying crop rotations can also reduce or eliminate crop yield reductions in zero-tillage systems relative to tilled systems [46, 47]. Zero-tillage systems have been recognized broadly for their positive contribution on soil conservation and fuel use efficiency [48]. Our results, coupled with those mentioned above, suggest synergies between zero-tillage and crop rotation in maintaining or improving crop yield and in suppressing weeds. Further research could examine in more detail how, and to what extent, different weed life history stages are affected by zero-tillage management within diverse rotations. In particular, more attention could be directed toward ways to optimize weed seed mortality in zero-tillage systems.

The remaining six moderators (increased crop species number, latitude classification, fallow inclusion in simple rotations, diversification from monoculture, herbicide usage, and perennial inclusion in diverse rotations) were not important for predicting weed responses in our dataset (Fig 4). As previously noted, crop species richness was much less important than crop-planting interval in explaining weed suppression. This demonstrates that the functional characteristics of diverse crop rotations are more important for determining weed responses than the number of crop species. Latitude’s lack of influence suggests that the effects of crop diversification on weeds are consistent across a wide range of environmental conditions. Our results also showed that replacing fallow in simple rotations with a more diverse crop rotation does not compromise weed control. The minimal influence of whether diversification occurred from a monoculture suggests that our detection of a diversification effect on weed density was not an artifact of including extremely simple cropping systems in our analysis.

The lack of importance of herbicide use for determining the response of weed density to rotational diversification is of particular interest. Herbicides have become the dominant tool for weed management in most modern agricultural systems. Doucet et al. (1999) showed that the suppressive effects of rotation diversification could be erased in the presence of strong management filters, such as herbicides [49]. While this phenomenon was observed for that study in particular, our results, which consider studies from a broader geographical extent, indicate that the weed suppressive effect offered by crop rotational diversification is evident irrespective of herbicide use. This finding supports the use of diverse rotations as an important practice in weed management irrespective of ancillary practices and management preferences. It also suggests that diverse rotations may become increasingly important for weed control as more herbicides lose efficacy due to evolving weed resistances.

Inclusion of perennials has been suggested as an important strategy within integrated weed management systems [13–15, 50]. However, in our BRT analysis, this moderator had minimal importance (Fig 4). Nonetheless, given the positive contribution of perennial forages to soil properties and hydrological processes [51, 52], additional work on how rotations that include perennials affect weed seed dynamics would be beneficial. Additionally, due to the large amount of variability we observed in weed responses to perennial inclusion, a more thorough examination of how other management factors interact (fertilization, duration of perennial crop growth, disturbance events, such as mowing and forage harvest) is warranted.

## Conclusions

Our results are consistent with the findings of prior qualitative reviews [15, 16] that indicate diverse rotations are more effective in suppressing weeds relative to simpler ones. However, our results paint a more nuanced picture. Diverse rotations may impact processes affecting weed density, such as weed seed germination and seedling mortality, but diversification may not be as powerful in limiting the growth of established weed seedlings. Other management practices, such as targeted herbicide or mechanical intervention, altered crop sowing density or row spacing configurations, or the selection of more competitive crop genotypes [10, 53], may play a stronger role in suppressing weed biomass. Additional studies measuring the effects of crop rotational diversity on weed biomass are needed. Based on our findings, rotational diversification strategies should carefully consider how to maximize temporal variance around crop planting dates. Implementing effective crop rotations that optimize planting-interval variation will entail site-specific considerations, and will require an amalgam of extension and outreach services, farmer-to-farmer learning communities, economic incentives, expanded market opportunities, and well-conceived regulatory mechanisms. The diversification of crop rotations can, and should, serve as an organizing principle, under which technological innovations and ecological insights can be joined to manage weeds and contribute to the sustainable management of agricultural systems.

## Supporting information

S1 Text Full list of studies included in the meta-analysis (n = 54). (PDF)

S2 Text Data extraction. (PDF)

S1 Fig PRISMA flow diagram of screening process for studies included in meta-analysis. (PDF)

S2 Fig Effect sizes over time. (PDF)

S1 Table PRISMA checklist for meta-analyses and systematic reviews. (PDF)

S1 Tables Statistical summaries. (PDF)
