# Supplementary material for: Does diversifying crop rotations suppress weeds? A meta-analysis
Source: PLoS One. 2019 Jul 18;14(7):e0219847. doi: 10.1371/journal.pone.0219847 (PMC6638938; doi:10.1371/journal.pone.0219847)
Supplement: S1 Text — (PDF) [file pone.0219847.s001.pdf]

S1 Text. Full list of studies included in the meta-analysis (n = 54).

Adamiak, E, Adamiak, J. Zachwaszczenie owsa w warunkach zróżnicowanego następstwa roślin i chemicznej ochrony łanu. Acta Scientifica Polonia Agricultura. 2004. 31: 119–28.

Anderson, RL. Crop sequence and no-till reduce seedling emergence of common sunflower (*Helianthus annuus*) in following years. Weed Technology. 2007 Jun; 21: 355–58.

Anderson, RL, Beck, DL. Characterizing weed communities among various rotations in central South Dakota. Weed Technology. 2007 Mar; 21: 76–9.

Anderson, RL, Stymiest, CE., Swan, BA, Rickertsen, JR. Weed community response to crop rotations in western South Dakota. Weed Technology. 2007 Mar; 21: 131–35.

Anderson, RL, Tanaka, DL, Black, AL, Schweizer, EE. Weed community and species response to crop rotation, tillage, and nitrogen fertility. Weed Technology. 1998 Jul; 12: 531–36.

Ball DA, Miller SD. Cropping history, tillage, and herbicide effects on weed flora composition in irrigated corn. Agronomy Journal. 1993; 85: 817-21.

Benoit, DL. Influence of carrot/onion/barley cropping sequence on the weed seed bank and field flora in an organic soil in Quebec, Canada. Aspects of Applied Biology. 2003. 69: 69–75.

Blackshaw, RE, Larney, FJ, Lindwall, CW, Watson, PR, Derksen, DA. Tillage intensity and crop rotation affect weed community dynamics in a winter wheat cropping system. Canadian Journal of Plant Science. 2001 Oct; 81: 805–13.

Blackshaw, RE. Rotation affects downy brome (*Bromus tectorum*) in winter wheat (*Triticum aestivum*). Weed Technology. 1994 Dec; 8: 728–32.

Blackshaw, RE., Larney, FO, Lindwall, CW, Kozub, GC. Crop rotation and tillage effects on weed populations on the semi-arid Canadian prairies. Weed Technology. 1994 Jun; 8: 231–237.

Blackshaw RE, Pearson DC, Larney FJ, Regitnig PJ, Nitschelm JJ, Lupwayi NZ. Conservation management and crop rotation effects on weed populations in a 12-year irrigated study. Weed Technology. 2015 Dec; 29: 835-43.

Buhler, DD., Stoltenberg, DE, Becker, RL, Gunsolus, JL. Perennial weed populations after 14 years of variable tillage and cropping practices. Weed Science. 1994 Jun; 42: 205–09.

Cathcart, R. J. et al. Rotation length, canola variety and herbicide resistance system affect weed populations and yield. Weed Science. 2006 Aug; 54: 726–34.

Chikoye, D, Ekeleme, F, Lum, AF, Schulz, S. Legume-maize rotation and nitrogen effects on weed performance in the humid and subhumid tropics of West Africa. Crop Protection. 2008 Mar; 27: 638–47.

Clay SA, Aguilar I. Weed seedbanks and corn growth following continuous corn or alfalfa. Agronomy Journal. 1998; 90: 813-8.

Covarelli, G, Tei F. Effet de la rotation culturale sur la flore adventice du maïs. VIIIème colloque international sur la biologie, l'écologie et la systématique des mauvaises herbes. 1988. 2: 477-84.

Daugovish O, Lyon DJ, Baltensperger DD. Cropping systems to control winter annual grasses in winter wheat (*Triticum aestivum*). Weed Technology. 1999 Jan; 1:120-6.

Davis VM, Gibson KD, Bauman TT, Weller SC, Johnson WG. Influence of weed management practices and crop rotation on glyphosate-resistant horseweed (*Conyza canadensis*) population dynamics and crop yield-years III and IV. Weed Science. 2009 Aug; 57: 417-26.

Demjanová E, Macák M, Dalovic I, Majernik F, Tyr S, Smatana S. Effects of tillage systems and crop rotation on weed density, weed species composition and weed biomass in maize. Agronomy research. 2009; 7: 785-92.

Doucet C, Weaver SE, Hamill AS, Zhang J. Separating the effects of crop rotation from weed management on weed density and diversity. Weed science. 1999 Nov; 1: 729-35.

Eiszner, H, Blandon, V, Pohlan, J. Rotación de cultivos en algodón - impactos agronómicos y ecológicos. Der Tropenlandwirt. 1996. 75–83.

Filizadeh H, Rezazadeh A, Younessi Z. Effects of crop rotation and tillage depth on weed competition and yield of rice in the paddy fields of Northern Iran. Journal of Agricultural Science and Technology. 2010 Jan 28; 9: 99-105.

Goplen JJ, Sheaffer CC, Becker RL, Coulter JA, Breitenbach FR, Behnken LM, Johnson GA, Gunsolus JL. Seedbank depletion and emergence patterns of giant ragweed (*Ambrosia trifida)* in Minnesota cropping systems. Weed Science. 2017 Jan; 65: 52-60.

Gulden RH, Sikkema PH, Hamill AS, Tardif F, Swanton CJ. Conventional vs. glyphosate-resistant cropping systems in Ontario: weed control, diversity, and yield. Weed Science. 2009 Dec; 57: 665-72.

Harker, KN, O'Donovan, JT, Irvine, RB, Turkington, TK, Clayton, GW. Integrating cropping systems with cultural techniques augments wild oat (*Avena fatua*) management in barley. Weed Science. 2009 Jun; 57: 326–37.

Harker KN, O'Donovan JT, Turkington TK, Blackshaw RE, Lupwayi NZ, Smith EG, Dosdall LM, Hall LM, Kutcher HR, Willenborg CJ, Peng G. Canola cultivar mixtures and rotations do not mitigate the negative impacts of continuous canola. Canadian Journal of Plant Science. 2015 Nov; 95: 1085-99.

Hunt, ND, Hill, JD, Liebman, M. Reducing freshwater toxicity while maintaining weed control, profits, and productivity: effects of increased crop rotation diversity and reduced herbicide usage. Journal of Environmental Science and Technology. 2017 Jan; 51: 1707–17.

Koocheki A, Nassiri M, Alimoradi L, Ghorbani R. Effect of cropping systems and crop rotations on weeds. Agronomy for sustainable development. 2009 Jun; 29:401-8.

Légère A, Stevenson FC, Benoit DL, Samson N. Seedbank–plant relationships for 19 weed taxa in spring barley – red clover cropping systems. Weed science. 2005 Oct; 53: 640-50.

Leroux GD, Benoît DL, Banville S. Effect of crop rotations on weed control, *Bidens cernua* and *Erigeron canadensis* populations, and carrot yields in organic soils. Crop Protection. 1996 Mar; 15: 171-8.

Liebman M, Miller ZJ, Williams CL, Westerman PR, Dixon PM, Heggenstaller A, Davis AS, Menalled FD, Sundberg DN. Fates of *Setaria faberi* and *Abutilon theophrasti* seeds in three crop rotation systems. Weed Research. 2014 Jun; 54: 293-306.

Manley BS, Wilson HP, Hines TE. Weed management and crop rotations influence populations of several broadleaf weeds. Weed Science. 2001 Feb; 49: 106-22.

Marenco RA, Santos ÁM. Crop rotation reduces weed competition and increases chlorophyll concentration and yield of rice. Pesquisa Agropecuária Brasileira. 1999 Oct; 34: 1881-7.

Martin RJ, Felton WL. Effect of crop rotation, tillage practice, and herbicides on the population dynamics of wild oats in wheat. Australian Journal of Experimental Agriculture. 1993; 33: 159-65.

Melander B, Holst N, Jensen PK, Hansen EM, Olesen JE. *Apera spica-venti* population dynamics and impact on crop yield as affected by tillage, crop rotation, location and herbicide programmes. Weed Research. 2008 Feb; 48: 48-57.

Mock VA, Creech JE, Ferris VR, Faghihi J, Westphal A, Santini JB, Johnson WG. Influence of winter annual weed management and crop rotation on soybean cyst nematode (*Heterodera glycines*) and winter annual weeds: years four and five. Weed Science. 2012 Dec; 60: 634-40.

Chamanabad HM, Ghorbani A, Asghari A, Tulikov AM, Zargarzadeh F. Long-term effects of crop rotation and fertilizers on weed community in spring barley. Turkish Journal of Agriculture and Forestry. 2009 Oct; 33: 315-23.

Porter PM, Huggins DR, Perillo CA, Quiring SR, Crookston RK. Organic and other management strategies with two-and four-year crop rotations in Minnesota. Agronomy Journal. 2003 Mar; 95: 233-44.

Rey-Caballero J, Royo-Esnal A, Recasens J, González I, Torra J. Management options for multiple herbicide–resistant corn poppy (*Papaver rhoeas*) in Spain. Weed Science. 2017 Mar; 65: 295-304.

Santín-Montanyá MI, Martín-Lammerding D, Zambrana E, Tenorio JL. Management of weed emergence and weed seed bank in response to different tillage, cropping systems and selected soil properties. Soil and Tillage Research. 2016 Aug; 161: 38-46.

Schreiber MM. Influence of tillage, crop rotation, and weed management on giant foxtail (*Setaria faberi*) population dynamics and corn yield. Weed Science. 1992 Dec; 40: 645-53.

Seibutis V, Deveikyte I. The influence of short crop rotations on weed community composition. Agronomy Research. 2006; 4: 353-7.

Shahzad M, Farooq M, Jabran K, Hussain M. Impact of different crop rotations and tillage systems on weed infestation and productivity of bread wheat. Crop protection. 2016 Nov; 89: 161-9.

Simić M, Spasojević I, Kovacević D, Brankov M, Dragicević V. Crop rotation influence on annual and perennial weed control and maize productivity. Romanian Agricultural Research. 2016; 33: 125-32.

Singer JW, Cox WJ, Hahn RR, Shields EJ. Cropping system effects on weed emergence and densities in corn. Agronomy Journal. 2000 Jul; 92: 754-60.

Soon YK, Darwent AL. Effect of integrated management of couch grass (*Elytrigia repens*) on soil quality and crop nutrition. The Journal of Agricultural Science. 1998 May; 130: 323-8.

Sosnoskie LM, Herms CP, Cardina J, Webster TM. Seedbank and emerged weed communities following adoption of glyphosate-resistant crops in a long-term tillage and rotation study. Weed Science. 2009 Jun; 57: 261-70.

Stevenson FC, Légère A, Simard RR, Angers DA, Pageau D, Lafond J. Weed species diversity in spring barley varies with crop rotation and tillage, but not with nutrient source. Weed Science. 1997 Nov: 798-806.

Taa A, Tanner D, Bennie AT. Effects of stubble management, tillage and cropping sequence on wheat production in the south-eastern highlands of Ethiopia. Soil and Tillage Research. 2004 Mar; 76: 69-82.

VanGessel MJ, Forney DR, Conner M, Sankula S, Scott BA. A sustainable agriculture project at Chesapeake Farms: a six-year summary of weed management aspects, yield, and economic return. Weed Science. 2004 Oct; 52: 886-96.

White AD, Stahlman PW, Northam FE. Impact of integrated management systems on jointed goatgrass (*Aegilops cylindrica*) populations. Weed science. 2004 Dec; 52: 1010-7.

Woźniak A, Soroka M. Structure of weed communities occurring in crop rotation and monoculture of cereals. International Journal of Plant Production. 2015 Jul; 9: 487-506.

Woźniak, A. The dynamics of sowing spring barley grown in various stands. Ann. Univ. Marie-Curie-Sklodowka. 2003; 58.

Young, FL, Ogg, AG Jr, Thill, DC, Young, DL, Papendick, RI. Weed management for crop production in the Northwest wheat (*Triticum aestivum*) region. Weed Science. 1996 Apr; 44: 429–436.
