# Supplementary material for: Does diversifying crop rotations suppress weeds? A meta-analysis
Source: PLoS One. 2019 Jul 18;14(7):e0219847. doi: 10.1371/journal.pone.0219847 (PMC6638938; doi:10.1371/journal.pone.0219847)
Supplement: S2 Text — (PDF) [file pone.0219847.s002.pdf]

S2 Text for Manuscript 'Does crop diversification supress weeds? A meta-analysis

Data extraction

The following table presents the data extracted from the studies listed in the meta-analysis bibliography. Each variable is discussed in more detail below. The raw data set, a complete data dictionary, and associated information are freely available through Iowa State University's DataShare wesbite. The citation for this dataset is as follows:

Nichols, Virginia, Weisberger David, Liebman Matt (2019). Effect of crop rotation on weed biomass and density literature summary. Iowa State University. Dataset. httpsL//doi.org/10.25380/iastate.7771010.v1

Table 1: Data extracted from published studies

Variable label	Variable name	Measurement unit
study_no	study number	categorical number
reference	short-hand notation for reference	categorical text
lat	latitude	decimal format degrees
long	longitude	decimal format degrees
data_type	data type with respect to weed sampling	categorical text
reps	replicates	count
sys_tillage	tillage classification of study	categorical text
sys_weedmgmt	type of weed management used in study	categorical text
weedmsmt_unit	what population unit the weed measurements were done on	categorical text
perenYN	perennial inclusion	binary
fallowYN	presence of fallow year	binary
checkcrop	check crop	crop name
cropseq	rotation crop sequence	crop name
rotlen_yrs	crop rotation length	years
species_nu	number of crop species in rotation	number
PICV	planting-interval-coefficient-of-variation	number
studyage_yrs	study age	years
weed_bio	weed biomass	grams per square meter
weed_bioSE	standard error of weed biomass value	grams per square meter
weed_den	weed density	plants per square meter
weed_denSE	standard error of weed density value	plants per square meter

lat and long, latitude class

For latitude, any study conducted above 35 deg latitude was considered temperate, while any study conducted below this latitude was considered sub-tropical or tropical (Fig. 1 of main manuscript). Studies were divided along these lines in order to have adequate sample sizes for analysis.

data_type and reps

If a study provided weed response data for multiple time points within a given site-year, data from the last reported measurement were used. Data were also reported in three different ways with respect to measurements over site-years: at an end time point (last, n = 110), averaged over multiple years (average, n = 61), or values from multiple years (repeated measures, n = 720). To create a data set with as much uniformity as possible, we transformed repeated measures data points into last and average response data

points based on the length of the study in relation to the length of the longest rotation. If data were measured after the study had been in place more than two rotation cycles, the responses were averaged, and the number of replicates associated with the data were adjusted accordingly. For data measured in studies less than two rotation cycles old, the last year's measurement was used. Several studies included treatments not relevant to our hypotheses (e.g. crop planting date, herbicide rate). In these instances, data were pooled over levels of the factor with no increase in effective replication.

sys_tillage and sys_weedmgmt

Data relative to the tillage regime and herbicide use were only extracted and included if applied uniformly over rotation treatments. Where tillage or herbicide treatments differed by rotation, these modifiers were assigned *IC* values, which were interpreted as *NAs* for analyses.

weedmsmt_unit

Where only individual weed species were studied, response data represent measurements of that species solely (single species). Where weed communities were studied, weed response data were aggregated (community).

fallowYN

Use of a fallow period in a rotation was recorded.

perenYN

Use of a perennial crop that was in place for more than one calendar year was recorded.

checkcrop

The crop the weed responses were measured in was recorded. Comparisons between rotations were only made if measurements were made in the same crop.

species_nu

For all rotations, the number of species 'units' present in the rotation were recorded. When calculating the number of species units present in a rotation, cover crops and pasture were counted as one species unit even if they were present as diverse mixtures composed of two or more species. We felt it was inappropriate to inflate the species number of a rotation if they were being grown together as one functional unit.

PICV

Planting month data were used in the development of an index accounting for the variance around planting date, a potential proxy for the functional diversity of a crop rotation system (Gaba et al., 2014). When information about the planting date of each crop in the study was not reported and was unavailable via personal communication, we used gray literature (extension and non-governmental organization publications) from institutions most proximate to study locations. The number of months between plantings for one complete rotation cycle was recorded, and the mean and standard deviation of those numbers was calculated. The coefficient of variation was calculated by dividing the standard deviation by the mean. This value was reported for each rotation.

monoYN

An additional modifier was assigned to comparisons of simple and diverse rotations that indicated whether the simple rotation was a monoculture. Any rotation with a species unit number of one was assigned monoculture status.
