# Supplementary figures and images for: Does diversifying crop rotations suppress weeds? A meta-analysis
Source: PLoS One. 2019 Jul 18;14(7):e0219847. doi: 10.1371/journal.pone.0219847 (PMC6638938; doi:10.1371/journal.pone.0219847)

**S1 Fig.** PRISMA flow diagram of screening process for studies included in meta-analysis.

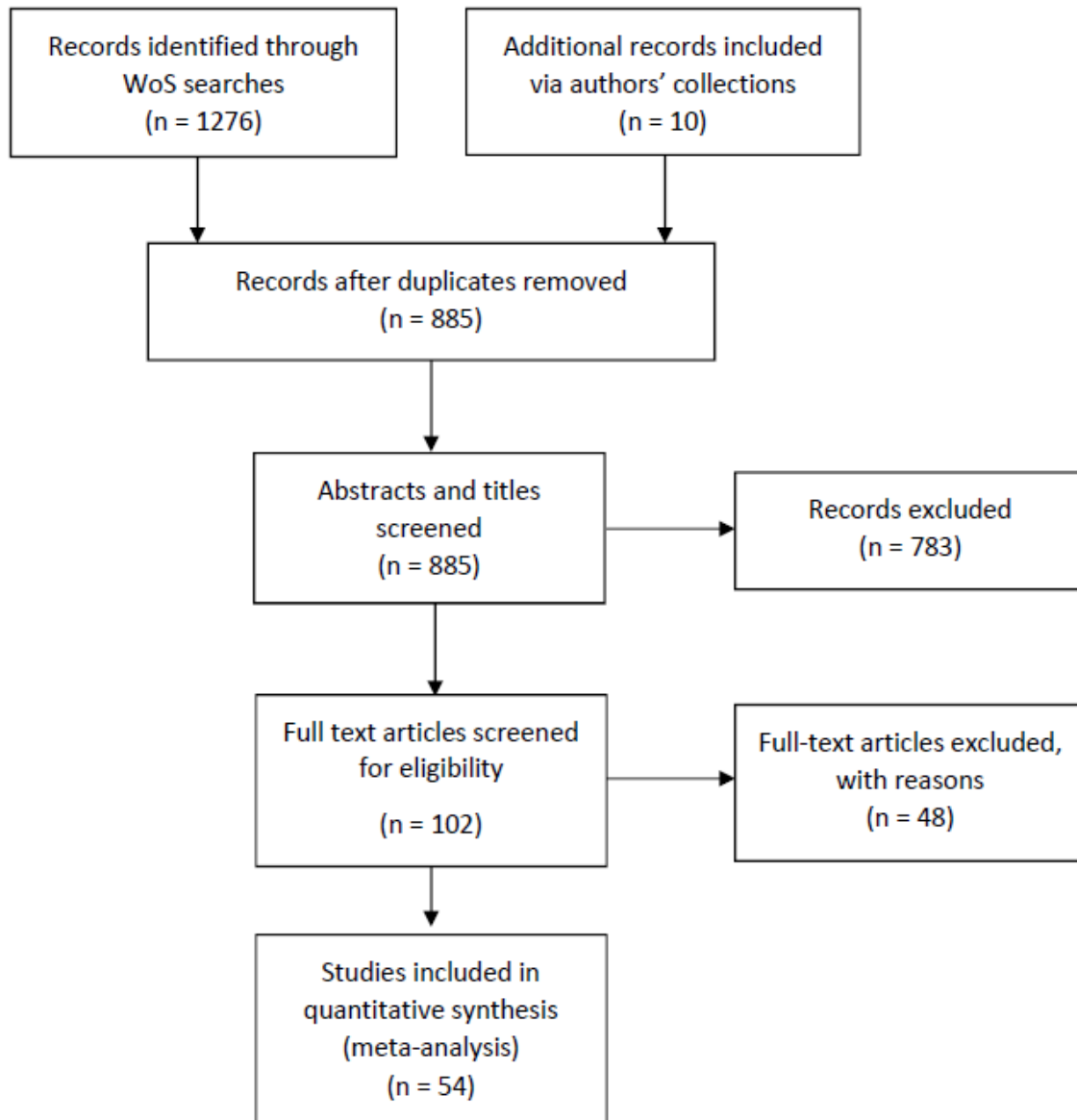

Supplement: S1 Fig — (PDF) [file pone.0219847.s003.pdf]
