# Supplementary material for: Does diversifying crop rotations suppress weeds? A meta-analysis
Source: PLoS One. 2019 Jul 18;14(7):e0219847. doi: 10.1371/journal.pone.0219847 (PMC6638938; doi:10.1371/journal.pone.0219847)
Supplement: S2 Fig — (PDF) [file pone.0219847.s004.pdf]

S2 Fig for Manuscript 'Does crop diversification supress weeds? A meta-analysis

Effect sizes over time

Effect sizes over time were investigated to look at trends in effect size and precision of estimates. Due to fewer studies, weed biomass responses are not known to the same precision as weed density response.

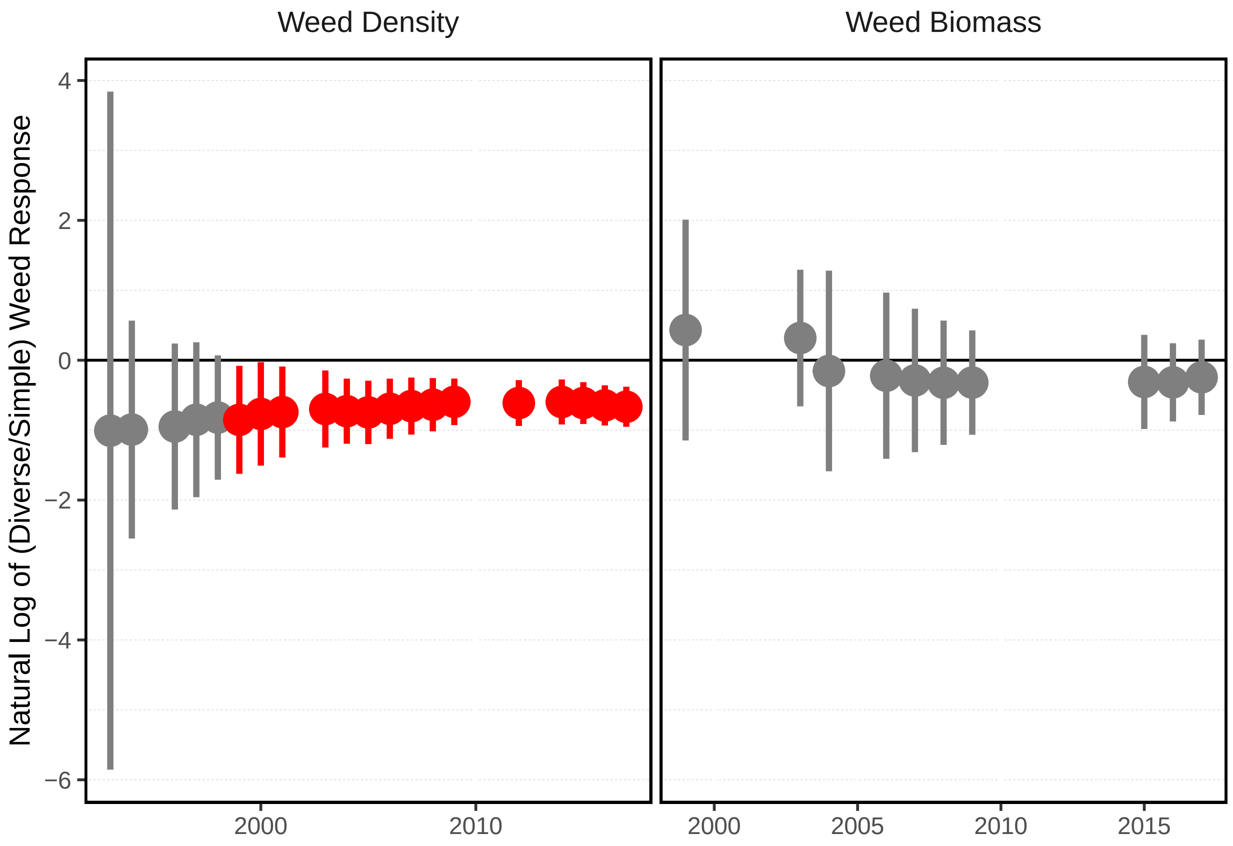

Figure 1: Effect size and 99% confidence intervals for accumulated studies by year, red indicates estimate is significantly less than 0
