# Supplementary material for: Does diversifying crop rotations suppress weeds? A meta-analysis
Source: PLoS One. 2019 Jul 18;14(7):e0219847. doi: 10.1371/journal.pone.0219847 (PMC6638938; doi:10.1371/journal.pone.0219847)
Supplement: S1 Tables — (PDF) [file pone.0219847.s006.pdf]

S1 Tables for Manuscript 'Does crop diversification supress weeds? A meta-analysis

Statistical summaries

All data and code for producing these summaries is available on github at https://github.com/vanichols/Weisberger-et-al-2019

Means, standard errors of the mean (SEM), Satterthwaite degrees of freedom (df), t-value, 99% confidence interval (CI) bounds and p-values are presented. Effects were determined to be significant at alpha = 0.01.

Table 1: Weed density and biomass estimates

Moderator	Estimate	SEM	df	t	Lower 99%CI	Upper 99%CI	p-value
Density	-0.666	0.107	51	-6.217	-0.952	-0.379	$<$0.001
Biomass	-0.244	0.179	13	-1.368	-0.783	0.294	0.195

Table 2: Leave-one-out sensitivity analysis summary for overall estimates

Response	Minimum 99% CI Value	Maximum 99% CI Value
Biomass	-0.867	0.384
Density	-0.984	-0.348

Table 3: Weed density moderator level significances

**Moderator**	**Level**	**Estimate**	**SEM**	**df**	**p-value**	**Lower 99%CI**	**Upper 99%CI**
Weed Unit	community	-0.480	0.110	79	<0.001	-0.771	-0.189
	single species	-1.216	0.192	141	<0.001	-1.718	-0.713
Tillage	NT	-1.049	0.178	242	<0.001	-1.510	-0.587
	tilled	-0.520	0.118	86	<0.001	-0.830	-0.210
Latitude	sub/tropical	-1.151	0.312	92	<0.001	-1.971	-0.331
	temperate	-0.601	0.114	83	<0.001	-0.901	-0.301
Simple System Fallow	N	-0.723	0.119	88	<0.001	-1.035	-0.410
	Y	-0.325	0.267	141	0.226	-1.022	0.373
Perennial Inclusion	N	-0.702	0.115	86	<0.001	-1.006	-0.398
	Y	-0.431	0.276	136	0.12	-1.152	0.289
Weed Control	herb_N	-0.682	0.165	155	<0.001	-1.113	-0.252
	herb_Y	-0.696	0.121	104	<0.001	-1.012	-0.379

Table 4: Weed biomass moderator level significances

**Moderator**	**Level**	**Estimate**	**SEM**	**df**	**p-value**	**Lower 99%CI**	**Upper 99%CI**
Weed Unit	community	-0.189	0.197	19	0.35	-0.753	0.375
	single species	-0.695	0.560	30	0.224	-2.235	0.844
Tillage	NT	-0.927	0.376	99	0.015	-1.914	0.060
	tilled	-0.163	0.185	22	0.389	-0.684	0.359
Latitude	sub/tropical	-0.323	0.341	17	0.356	-1.309	0.663
	temperate	-0.211	0.228	21	0.366	-0.858	0.436
Simple System Fallow	N	-0.223	0.197	20	0.27	-0.783	0.336
	Y	-0.554	0.714	10	0.456	-2.824	1.716
Perennial Inclusion	N	-0.392	0.163	20	0.026	-0.855	0.072
	Y	0.779	0.414	60	0.065	-0.323	1.882
Weed Control	herb_N	-0.203	0.275	31	0.466	-0.956	0.551
	herb_Y	-0.294	0.227	26	0.208	-0.925	0.337

Table 5: Leave-one-out sensitivity analysis results, moderators

Response	Moderator	Level	Minimum 99% CI Value	Maximum 99% CI Value
Density	climclasslat2	sub/tropical	-2.285	-0.071
		temperate	-0.937	-0.263
	fallow__simpYN	N	-1.070	-0.322
		Y	-1.592	0.584
	peren__divYN	N	-1.044	-0.364
		Y	-1.340	0.436
	sys__tillage	NT	-1.591	-0.469
		tilled	-0.868	-0.151
	sys__weedmgmt2	herb__N	-1.441	-0.149
		herb__Y	-1.053	-0.328
	weedmsmt__unit	community	-0.807	-0.149
		single species	-1.835	-0.573
Biomass	climclasslat2	sub/tropical	-1.581	0.915
		temperate	-1.002	0.567
	peren__divYN	N	-0.915	0.163
		Y	-0.906	2.828
	sys__tillage	NT	-2.787	0.468
		tilled	-0.761	0.457
	sys__weedmgmt2	herb__N	-1.225	0.876
		herb__Y	-1.123	0.490
	weedmsmt__unit	community	-0.848	0.478
		single species	-3.429	1.637

Table 6: Comparisons of moderator level estimates

Response	Moderator	level1	level2	Estimate	SEM	df	Statistic	p-value
Density	Weed Unit	community	single species	0.735	0.222	121	3.316	0.001
	Tillage	NT	tilled	-0.528	0.178	769	-2.972	0.003
	Latitude	sub/tropical	temperate	-0.550	0.332	91	-1.657	0.101
	Simple System Fallow	N	Y	-0.398	0.282	162	-1.411	0.160
	Perennial Inclusion	N	Y	-0.271	0.293	145	-0.923	0.357
	Weed Control	herb_N	herb_Y	0.013	0.163	566	0.082	0.935
Biomass	Weed Unit	community	single species	0.506	0.594	28	0.853	0.401
	Tillage	NT	tilled	-0.765	0.368	125	-2.077	0.040
	Latitude	sub/tropical	temperate	-0.112	0.410	18	-0.273	0.788
	Simple System Fallow	N	Y	0.331	0.741	10	0.446	0.665
	Perennial Inclusion	N	Y	-1.171	0.445	50	-2.630	0.011
	Weed Control	herb_N	herb_Y	0.091	0.300	89	0.303	0.762

Table 7: Moderator relative influence as estimated by a generalized boosted regression model

Moderator	Relative Influence
weedmsmt_unit	35.36
PICV_diff	26.73
sys_tillage	23.70
climclasslat2	6.19
sys_weedmgmt2	3.97
species_rat	1.55
peren_divYN	1.35
fallow_simpYN	1.05
mono_YN	0.10
